# The relationship between different anticoagulation monitoring indicators and heparin doses in adult patients with extracorporeal cardiopulmonary resuscitation

**DOI:** 10.3389/fcvm.2025.1653774

**Published:** 2025-12-02

**Authors:** Jiali Yao, Cong Jin, Tianxing Du, Huizhen Yu

**Affiliations:** 1 Shengli Clinical Medical College of Fujian Medical University, Fujian, China; 2 Department of Critical Care Medicine, Jinhua Hospital Affiliated to Zhejiang University, Zhejiang, China; 3 Department of Breast and Thyroid Surgery, Jinhua Hospital Affiliated to Zhejiang University, Zhejiang, China; 4 Department of Geriatric Medicine, Fuzhou University Affiliated Provincial Hospital, Fujian, China

**Keywords:** extracorporeal membrane oxygenation, heparin, activated clotting time, activated partial thromboplastin time, anticoagulant

## Abstract

**Objectives:**

Achieving optimal systemic anticoagulation in patients undergoing extracorporeal membrane oxygenation (ECMO) requires precise titration, posing a significant clinical challenge. This study aims to explore the relationship between various anticoagulation monitoring parameters and heparin dosing in adult patients undergoing extracorporeal cardiopulmonary resuscitation (ECPR).

**Methods:**

A retrospective analysis was conducted on 40 ECPR patients, who were categorized into either the activated clotting time (ACT)-target group or the activated partial thromboplastin time (aPTT)-target group. Anticoagulation monitoring parameters, including aPTT, corresponding ACT, and heparin doses, were collected. Continuous variables are expressed as mean ± SD and compared using Student's *t*-test. Pearson correlation analysis was performed to examine the relationships between ACT, aPTT, and heparin doses in both groups.

**Results:**

Of the 40 patients, 16 (40%) died and 24 (60%) survived. Twelve patients (30%) experienced severe bleeding events, while five (12.5%) developed thrombotic complications. Compared with the ACT-target group, the aPTT-target group had higher platelet counts (PLT) and lower blood flow levels (*P* < 0.001). Pearson correlation analysis revealed that heparin dose was positively correlated with aPTT (*r* = 0.412, *P* < 0.001) and negatively correlated with blood flow (*r* = −0.329, *P* < 0.001). ACT showed a weak correlation with aPTT (*r* = 0.123, *P* < 0.001).

**Conclusion:**

Heparin dosing exhibited a stronger correlation with aPTT than with ACT, and blood flow emerged as an important monitoring parameter. Comprehensive anticoagulation monitoring strategies should be employed to evaluate heparin efficacy in ECMO-treated ECPR patients.

## Introduction

In recent years, veno-arterial extracorporeal membrane oxygenation (VA-ECMO) has become increasingly utilized for extracorporeal cardiopulmonary resuscitation (ECPR), providing emergent support for patients with severe, reversible respiratory and cardiopulmonary failure (1). The interaction between ECMO components and blood induces inflammatory and thrombotic responses, necessitating anticoagulation to mitigate thrombotic risks (2). Unfractionated heparin (UFH) remains the primary anticoagulant, although its prolonged use heightens the risk of excessive bleeding (3). The key mechanism of heparin action involves its interaction with thrombin. UFH induces a conformational change in antithrombin (AT), thereby enhancing AT-mediated anticoagulation. Activated AT subsequently inhibits procoagulant factors, including thrombin, as well as factors IXa, Xa, XIa, and XIIa, which disrupts the conversion of fibrinogen to fibrin, thus preventing clot formation and extending blood clotting time (4). Despite its effectiveness, bleeding and thrombotic events continue to be significant complications associated with ECPR.

Many ECMO centers base anticoagulation management on activated clotting time (ACT), activated partial thromboplastin time (aPTT), or anti-Xa assay results. The Extracorporeal Life Support Organization (ELSO) advocates for the development of individualized anticoagulation protocols tailored to each center's clinical resources and patient population. Despite these recommendations, anticoagulation in ECMO remains challenging. ACT, which measures whole blood coagulation, is commonly used as a bedside test to guide UFH administration in ECMO patients. Studies have highlighted its advantages over laboratory tests, such as a faster turnaround time and the ability to be performed by non-laboratory staff (5). In contrast, aPTT is unaffected by platelet count or erythrocyte-specific volume and correlates well with heparin concentration. The aPTT assay offers sensitive monitoring of UFH levels within the 0.1–1 U/mL range, although higher UFH concentrations can cause aPTT to extend beyond the linear monitoring range (6). While ACT and aPTT are commonly employed to guide heparin dosing, the use of either test alone for anticoagulation management during ECMO has not been recommended for decades. A systematic review of 1,496 patients receiving VA-ECMO revealed major bleeding (27%) and thromboembolic complications (8%) as common adverse events, with limb ischemia, circuit thrombosis, and cerebrovascular events notably linked to anticoagulation monitoring strategies (7). Thus, maintaining a delicate balance in systemic anticoagulation titration for ECMO patients is critical. Optimal anticoagulation monitoring aims to minimize bleeding and thrombotic complications, ultimately enhancing management quality and reducing mortality and healthcare costs (8).

Currently, most hospitals continue to rely heavily on ACT and aPTT monitoring, although these assays may show variability across clinical centers, patient populations, and even within the same patient at different time points. Currently, there is a lack of literature evaluating the relationship between various anticoagulation assays and the maintenance heparin dose in VA-ECMO-treated adult ECPR patients. This study aims to explore the correlation between different anticoagulation monitoring parameters (such as ACT, aPTT, PT, and blood flow) and heparin dosing in adult ECPR patients, offering more precise guidance for clinical decision-making.

## Materials and methods

A single-center retrospective analysis was conducted on 58 adult patients who received VA-ECMO treatment between March 2023 and April 2025 in the intensive care unit (ICU) of Jinhua Hospital Affiliated with Zhejiang University. The inclusion criteria for the cohort were (i) VA-ECMO support for ≥72 h, (ii) age ≥18 years, and (iii) use of heparin for anticoagulation. The exclusion criteria included (i) age <18 years, (ii) death within 72 h, (iii) patients transferred from other hospitals, (iv) use of anticoagulants other than heparin or no heparin infusion during ECMO support, (v) missing data or fewer than 18 paired ACT and aPTT measurements, and (vi) pregnancy. Ultimately, 40 patients were included in the study. Given the retrospective nature of the research, written informed consent for participation was not required for the use of clinical specimens. The study adhered to the Declaration of Helsinki and received approval from the Ethics Committee of Jinhua Hospital Affiliated to Zhejiang University (Approval No. 2023-28).

### Blood collection and measurements

Patient characteristics assessed included age, sex, acute physiology, and chronic health evaluation II (APACHE II) score on admission, height, weight, and ECMO indications, which included fulminant myocarditis (FM), acute myocardial infarction (AMI), pulmonary embolism (PE), drug poisoning (DP), and others. Baseline laboratory values, including hemoglobin (Hb), creatinine (CR), blood urea nitrogen (BUN), platelet count (PLT), prothrombin time (PT), and international normalized ratio (INR), were measured within 6 h of ECMO initiation.

### Anticoagulation monitoring

According to the ELSO, an initial UFH bolus of 50–100 units per kilogram of body weight is recommended at the time of cannulation for ECMO, followed by continuous UFH infusion. The maintenance dose of UFH is adjusted based on ACT and aPTT results, with target ranges of 180–220 s for ACT and 60–80 s for aPTT. Patients were divided into either the ACT-target group or the aPTT-target group based on clinician discretion, informed by individual patient factors and clinical practice guidelines (9). In cases of bleeding, clinicians assessed the patient's clinical condition and adjusted heparin dosing, which may include reducing the dose or temporarily discontinuing anticoagulation. ACT was tested every 2 h, while aPTT was measured every 4 h, with additional tests conducted if there were changes in the ECMO circuit or the patient's clinical status. All patients received targeted temperature management, maintaining a target temperature of 34°C for at least 24 h before gradual rewarming. Anticoagulation monitoring, including aPTT, matched ACT, and heparin doses, was performed, with paired samples defined as those collected simultaneously or within 20 min of each other. Samples were excluded if the heparin infusion rate was adjusted between the collection of ACT and aPTT.

### Study endpoint

The primary endpoints of this study included the correlation between heparin dosage and both ACT and aPTT. Secondary endpoints encompassed patient outcomes and the relationship between various anticoagulation monitoring methods and mortality. Patient outcomes included ECMO duration, ICU stay, mortality, the need for continuous renal replacement therapy (CRRT), bleeding events, thrombotic complications, and blood transfusion volume.

Thromboembolic complications were defined as symptomatic events (e.g., stroke and leg ischemia) and circuit or oxygenator thromboses that disrupted ECMO function (e.g., reduced blood flow or impaired gas exchange).

Serious bleeding complications included bleeding at cannula insertion sites requiring intervention, gastrointestinal bleeding (GIB), hemorrhage from recent surgical sites, intracranial hemorrhage, and other organ bleeding necessitating blood transfusion.

### Statistical analysis

Statistical analyses were performed using IBM SPSS Statistics Version 25 (IBM Corporation, New York, NY, USA). Continuous variables with a normal distribution are presented as mean ± standard deviation, with comparisons made using Student's *t*-test. Categorical data are presented as frequencies and percentages, with Fisher's exact test used for analysis. Pearson correlation coefficients were calculated for correlation analyses. Scatter plots were generated using GraphPad Prism 10.1.2. A *P*-value of <0.05 was considered statistically significant.

## Results

### Baseline characteristicstics

**Figure 1:**
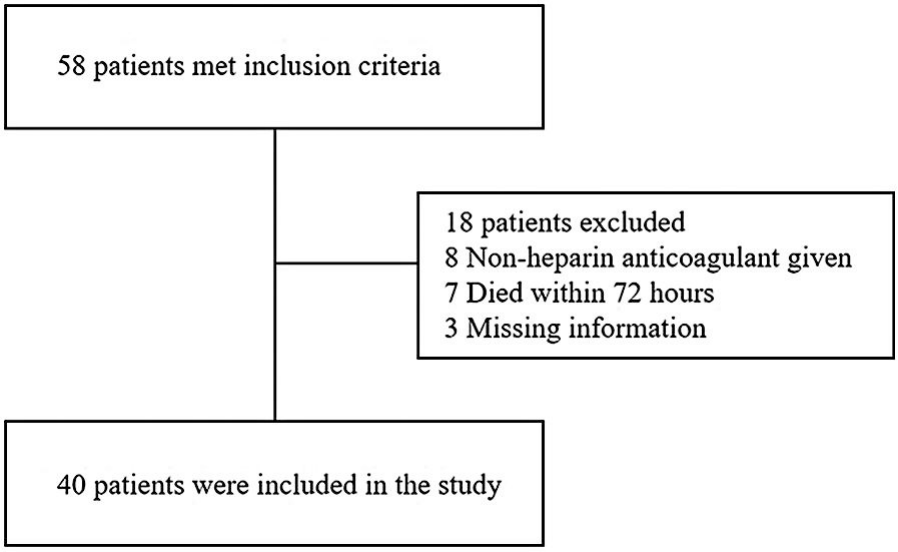
Flow diagram of patient enrollment in the study.

A total of 58 patients who received VA-ECMO after ECPR were identified during the study period, of which 40 patients met the inclusion criteria. Eight patients were excluded due to the absence of heparin administration, a primary exclusion criterion. Seven patients were excluded because they died within 72 h (Figure 1). Three patients were excluded due to missing data. Among the 40 included patients, 25 (62.5%) were male and 15 (37.5%) were female. Of these, 22 patients were assigned to the ACT-target group and 18 patients to the aPTT-target group. In the aPTT-target group, PLT levels were higher, while PT, INR, and PT/INR levels were lower compared with those in the ACT-target group. No significant differences were observed between the two groups in terms of age, gender, APACHE II score, height, weight, ECMO indications, or baseline Hb, CR, and BUN levels (Table 1).

**Table 1:** Baseline characteristics of the patients.

Variables	ACT target (*n* = 22)	aPTT target (*n* = 18)	*P*
Age (years)	46.10 ± 16.45	53.22 ± 19.52	0.179
Gender (male)	13 (59.09)	12 (66.67)	0.952
APACHE II score	31.62 ± 6.21	28 ± 5.34	0.064
Height (cm)	165.38 ± 7.65	164.28 ± 9.90	0.636
Weight (kg)	64.72 ± 9.18	64.89 ± 14.61	0.853
ECMO indications, *n* (%)			
FM	8 (36.4)	4 (22.22)	0.332
AMI	6 (27.3)	10 (55.56)	0.069
PE	4 (18.18)	3 (16.67)	0.900
DP	3 (13.64)	0 (0)	0.103
Others	1 (4.55)	1 (5.56)	0.884
Clinical measures			
HB (g/L)	111.19 ± 23.49	103.22 ± 16.02	0.242
PLT (×10^9^/L)	147.62 ± 58.68	164.89 ± 57.24	0.008*
PT (s)	17.83 ± 5.99	13.93 ± 3.14	0.013*
INR	1.72 ± 0.59	1.45 ± 0.33	0.028*
PT/INR	10.62 ± 1.91	9.78 ± 1.85	0.032*
CR (umol/L)	141.53 ± 68.11	167.78 ± 91.70	0.235
BUN (mmol/L)	9.99 ± 2.58	14.97 ± 13.57	0.133

APACHE II, Acute Physiology and Chronic Health Evaluation II; FM, fulminant myocarditis; AMI, acute myocardial infarction; PE, pulmonary embolism; DP, drug poisoning; HB, hemoglobin; PLT, platelet; PT, prothrombin time; INR, international normalized ratio; PT/INR: prothrombin time and international normalization ratio, CR, creatinine; BUN, blood urea nitrogen.

* *P* < 0.05.

### Patient outcomes

Of the 40 patients enrolled, 16 (40.0%) died during hospitalization, while 22 (55.0%) required CRRT. Twelve patients (30.0%) experienced serious bleeding events: in the ACT-target group, two patients had bleeding at cannula sites, four had GIB, and one had hemorrhage from another organ requiring blood transfusion; in the aPTT-target group, one patient had bleeding at cannula sites, three had GIB, and one had hemorrhage from another organ requiring transfusion. Five patients (12.5%) experienced thrombotic events: in the ACT-target group, one patient had a stroke, and two had leg ischemia; in the aPTT-target group, one patient had a stroke, and one had leg ischemia. Blood flow was lower in the aPTT-target group compared with that in the ACT-target group. No significant differences were observed between the two groups regarding ECMO duration, ICU stay, mortality at discharge, CRRT use, thrombotic events, serious bleeding events, heparin dose, ACT, aPTT, rotational speed, or volumes of red blood cell (RBC), fresh frozen plasma (FFP), and PLT transfusions (Table 2).

**Table 2:** Patient outcomes.

Variables	ACT target (*n* = 22)	aPTT target (*n* = 18)	*P*
Duration of ECMO (days)	6.36 ± 3.14	9.50 ± 10.56	0.087
ICU stay (day)	14.77 ± 10.85	14.44 ± 11.05	0.880
Mortality rate at discharge, *n* (%)	7 (31.8)	9 (50.0)	0.243
CRRT, *n* (%)	11 (50.0)	11 (61.1)	0.482
Adverse event, *n* (%)			
Thrombotic events, *n* (%)	3 (13.64)	2 (11.11)	0.810
Serious bleeding event, *n* (%)	7 (31.82)	5 (27.78)	0.781
Heparin dose (units/kg/hour)	10.50 ± 3.79	13.81 ± 2.44	0.648
ACT (s)	174.69 ± 40.03	179.45 ± 13.62	0.363
aPTT (s)	52.86 ± 16.69	50.42 ± 15.65	0.537
Blood flow (L/min)	2.93 ± 1.18	2.89 ± 0.65	0.025*
Rotational speed (rad/s)	3,015.82 ± 571.59	3,538.17 ± 1,588.11	0.071
RBC transfusion volume (U)	9.16 ± 8.13	14.17 ± 9.49	0.243
FFP transfusion volume (ml)	1,957.27 ± 1,060.87	1,675.56 ± 686.54	0.114
PLT transfusion volume (U)	2.98 ± 2.96	8.64 ± 3.77	0.090

ECMO, extracorporeal membrane oxygenation; ICU, intensive care unit; CRRT, continuous renal replacement therapy; ACT, activated clotting time; aPTT, activated partial thromboplastin time; RBC, red blood cell; FFP, fresh frozen plasma; PLT, platelet.

* *P* < 0.05.

### Correlation between the ACT and aPTT and the heparin dosage

**Figure 2:**
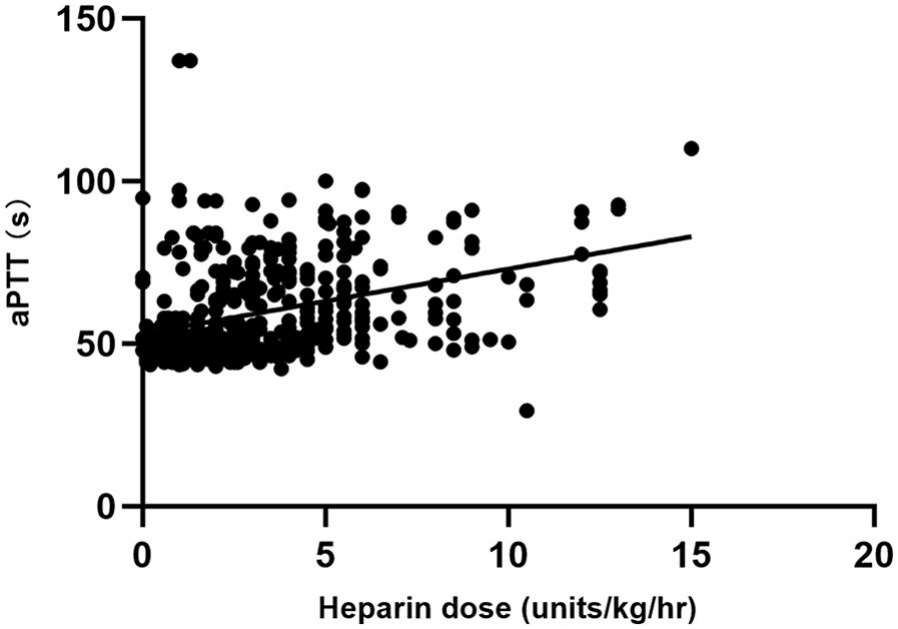
Comparison between heparin dosage and aPTT results.

**Figure 3:**
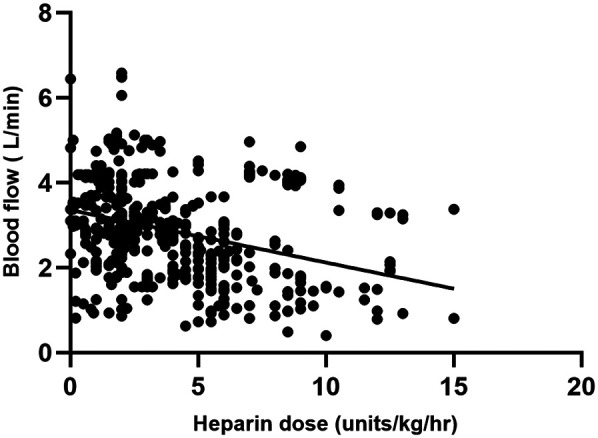
Comparison between heparin dosage and blood flow results.

A total of 446 matched pairs of ACT, aPTT, and heparin dose results were obtained from the 40 patients included in the study (22 in the ACT-target group and 18 in the aPTT-target group). Pearson correlation analysis was conducted to examine the relationships between matched ACT, aPTT, and heparin dosage. Heparin dose showed a positive correlation with aPTT (*r* = 0.412, *P* < 0.001) and a negative correlation with blood flow (*r* = −0.329, *P* < 0.001). ACT exhibited a weak correlation with aPTT (*r* = 0.123, *P* < 0.001), while no correlation was observed between heparin dose and ACT (Figures 2, 3).

When analyzing the correlations among ACT, aPTT, blood flow, and heparin doses during adult VA-ECMO in the two groups, it was found that in the ACT-target group, heparin dose was positively correlated with aPTT and negatively correlated with blood flow. ACT showed a weak correlation with aPTT, with no correlation to heparin dose. In the aPTT-target group, heparin dose was correlated with aPTT and negatively correlated with blood flow. ACT also showed weak correlations with both aPTT and heparin dose (Table 3).

**Table 3:** Correlation between ACT and aPTT and the heparin dosage.

Variables	ACT targe (*n* = 245)		aPTT target *(n* = 201)	
	*r*	*P*	*r*	*P*
Heparin dose and ACT	−0.042	0.407	−0.120	0.031*
Heparin dose and aPTT	0.417	0.001*	0.442	0.011*
Heparin dose and blood flow	−0.396	0.001*	−0.375	0.001*
ACT and aPTT	0.235	0.027*	0.249	0.001*

ACT, activated clotting time; aPTT, activated partial thromboplastin time.

* *P* < 0.05.

### The relationship between anticoagulation parameters and survival rate

Among the 40 enrolled patients, 16 (40%) died and 24 (60%) survived. Analysis of the relationship between anticoagulation parameters and survival rate revealed that ACT, blood flow, heparin dose, and PT were higher in the non-survival group, while PLT levels were lower compared with those in the survival group. No significant differences were observed in aPTT, APACHE II score, or INR between the two groups (Table 4).

**Table 4:** The relationship between anticoagulation parameters and survival rate.

Variables	Survival group (*n* = 24)	Non-survival group (*n* = 16)	*P*
ACT (s)	178.37 ± 12.35	182.35 ± 42.26	0.026*
aPTT (s)	47.73 ± 14.17	49.11 ± 17.95	0.474
Heparin dose (units/kg/h)	6.72 ± 2.79	8.88 ± 1.75	0.017*
Blood flow (L/min)	2.37 ± 0.69	3.45 ± 0.90	0.001*
PT (s)	15.92 ± 5.01	16.13 ± 5.42	0.007*
INR	1.55 ± 0.49	1.64 ± 0.51	0.555
PT/INR	10.03 ± 2.03	11.45 ± 1.80	0.286
PLT (×10^9^/L)	186.45 ± 79.32	137.25 ± 48.59	0.023*
APACHE II score	28.85 ± 5.82	30.95 ± 6.05	0.271

ACT, activated clotting time; aPTT, activated partial thromboplastin time; PT, prothrombin time; INR, international normalized ratio; PT/INR, prothrombin time and international normalization ratio; APACHE II, acute physiology and chronic health evaluation II.

* *P* < 0.05.

## Discussion

In this study, 16 patients (40.0%) died, 22 (55.0%) required CRRT, 12 (30.0%) experienced bleeding events, and 5 (12.5%) had thrombotic events. The aPTT-target group exhibited higher PLT and lower blood flow levels compared with those in the ACT-target group. However, no significant differences in mortality, CRRT requirement, blood transfusion, or bleeding and thrombotic events were found between the two groups. In the non-survival group, ACT, blood flow, heparin dose, and PT were higher, while PLT was lower compared with that in the survival group. Additionally, among the paired ACT, aPTT, and heparin dose data, heparin dose showed a positive correlation with aPTT and a negative correlation with blood flow. ACT was weakly correlated with aPTT and showed no correlation with heparin dose. During ECMO treatment of ECPR patients, heparin dose was more strongly correlated with aPTT than with ACT, and blood flow emerged as a potentially significant parameter.

Maintaining coagulation homeostasis remains a substantial challenge during ECMO support in ECPR patients, although it is crucial for improving clinical outcomes (10). In the present study, the aPTT-target group had higher PLT counts and lower blood flow, PT, INR, and PT/INR ratios compared with those in the ACT-target group. This suggests that patients in the aPTT-target group may exhibit better coagulation function, which could help reduce blood flow during ECMO. One study suggests that ECMO-related bleeding complications are associated with PT, INR, PT/INR, and low platelet count, with low platelet levels increasing the risk of death (11). However, the link between low platelet count and mortality shows population heterogeneity, with the ECMO population likely affected by mechanical consumption and coagulation disorders. Consequently, close monitoring of these parameters during ECMO support is critical. Furthermore, the analysis of anticoagulation parameters and survival rates revealed that in the non-survival group, ACT, blood flow, heparin dose, and PT were higher, while PLT was lower compared with that in the survival group.

Among the paired sample data for ACT, aPTT, and heparin dose, heparin dose exhibited a stronger correlation with aPTT than with ACT, with the relationship between aPTT and ACT being weak. In both groups, heparin dose was positively correlated with aPTT. A similar study by Liu et al. (12) also demonstrated that aPTT, rather than ACT, is more reliable for heparin dose adjustment when no superior monitoring indicators are available. Induced hypothermia therapy, often used alongside ECPR to improve neurological outcomes, can affect ACT test results, which may explain the poor correlation between ACT and heparin. Additionally, because aPTT is typically measured in plasma in the laboratory, its results are less affected by platelet count or hematocrit, unlike ACT. This may explain why aPTT correlates more reliably with heparin concentrations during ECMO support, although the correlation remains moderate. Thus, in complex anticoagulation scenarios such as ECMO, a single indicator may not fully or accurately reflect anticoagulation status, necessitating joint monitoring for more comprehensive evaluation. Although ELSO guidelines recommend both ACT and aPTT as monitoring tools, they do not specify an optimal anticoagulation target. Further research is needed to provide detailed guidance for optimal anticoagulation monitoring. Consequently, during the treatment of ECPR patients with ECMO, a comprehensive anticoagulation monitoring strategy should be employed to assess the efficacy of heparin.

Reducing ECMO blood flow rates is essential for safe weaning from VA-ECMO. One study demonstrated that low-flow ECMO (1.5 L/min) increases hemolysis, decreases platelet aggregation, and may exacerbate clot formation in the circuit oxygenator (13). A retrospective study involving 35 adult patients with VV-ECMO found that reducing ECMO blood flow by 1 L/min was associated with a daily haptoglobin consumption of 93.371 mg/dL, with haptoglobin serving as a sensitive marker for hemolysis (14). This aligns with our findings, where a negative correlation between heparin dose and blood flow was observed in both groups. A higher heparin dose may be necessary to achieve effective anticoagulation in low-flow conditions, likely due to the increased risk of thrombus formation.

Despite conventional coagulation tests falling within the target range, complications such as thromboembolism and bleeding can still occur. Excessive anticoagulation is associated with increased mortality during ECMO, and thrombosis can develop in the heart or major vasculature. In this study, no significant differences in mortality, CRRT requirement, or bleeding and thrombotic events were observed between the ACT-target and aPTT-target groups. Twelve patients (30.0%) experienced serious bleeding events, and five patients (12.5%) experienced thrombotic events. These findings are consistent with research by Mazzeffi et al. (15), which reported no apparent difference in the incidence of bleeding or thrombosis between VA-ECMO patients managed with either ACT or aPTT-guided heparin anticoagulation protocols. Therefore, it can be concluded that bleeding and thrombotic events are not significantly associated with the type of anticoagulation monitoring used. Additionally, patients who died within 72 h were excluded from this study, which may have resulted in a higher survival rate.

This study has several limitations. aPTT can be affected by hemolysis, high bilirubin, elevated lipids, and blood flow. While rotational speed may influence hemolysis, these data were not collected. Furthermore, the cause of death was not addressed in this study, preventing us from determining whether deaths were due to bleeding or thrombotic events. Future studies will incorporate this data to minimize this limitation. Additionally, the study's relatively small sample size, retrospective design, and single-center data highlight the need for larger multicenter studies to validate and generalize these findings.

## Conclusion

Heparin dose exhibited a stronger correlation with aPTT than with ACT, and blood flow may be an important monitoring parameter. During ECMO treatment for ECPR patients, comprehensive anticoagulation monitoring strategies should be used to evaluate the effectiveness of heparin, rather than relying solely on a single indicator for anticoagulation management.

## Data Availability

The raw data supporting the conclusions of this article will be made available by the authors, without undue reservation.
